# A Novel Whole-Cell Biocatalyst with NAD^+^ Regeneration for Production of Chiral Chemicals

**DOI:** 10.1371/journal.pone.0008860

**Published:** 2010-01-26

**Authors:** Zijun Xiao, Chuanjuan Lv, Chao Gao, Jiayang Qin, Cuiqing Ma, Zhen Liu, Peihai Liu, Lixiang Li, Ping Xu

**Affiliations:** 1 State Key Laboratory of Microbial Technology, Shandong University, Jinan, People's Republic of China; 2 MOE Key Laboratory of Microbial Metabolism and School of Life Sciences and Biotechnology, Shanghai Jiao Tong University, Shanghai, People's Republic of China; Universität Heidelberg, Germany

## Abstract

**Background:**

The high costs of pyridine nucleotide cofactors have limited the applications of NAD(P)-dependent oxidoreductases on an industrial scale. Although NAD(P)H regeneration systems have been widely studied, NAD(P)^+^ regeneration, which is required in reactions where the oxidized form of the cofactor is used, has been less well explored, particularly in whole-cell biocatalytic processes.

**Methodology/Principal Findings:**

Simultaneous overexpression of an NAD^+^ dependent enzyme and an NAD^+^ regenerating enzyme (H_2_O producing NADH oxidase from *Lactobacillus brevis*) in a whole-cell biocatalyst was studied for application in the NAD^+^-dependent oxidation system. The whole-cell biocatalyst with (2*R*,3*R*)-2,3-butanediol dehydrogenase as the catalyzing enzyme was used to produce (3*R*)-acetoin, (3*S*)-acetoin and (2*S*,3*S*)-2,3-butanediol.

**Conclusions/Significance:**

A recombinant strain, in which an NAD^+^ regeneration enzyme was coexpressed, displayed significantly higher biocatalytic efficiency in terms of the production of chiral acetoin and (2*S*,3*S*)-2,3-butanediol. The application of this coexpression system to the production of other chiral chemicals could be extended by using different NAD(P)-dependent dehydrogenases that require NAD(P)^+^ for catalysis.

## Introduction

Many approaches have been developed to produce chiral chemicals for the manufacture of a wide range of intermediates in the pharmaceutical, agrochemical, fine chemical and food industries [1]. In recent years, biocatalysis has emerged as a powerful strategy for the production of enantiomerically pure building blocks that cannot be obtained through chemical and fermentation processes [2]. NAD(P)-dependent oxidation systems have been used for the kinetic resolution of chiral alcohols and amines from a mixture of stereoisomers and for the production of ketones that are difficult to synthesize chemically [3]–[4]. However, due to the high costs of pyridine cofactors, an efficient cofactor regeneration system is a prerequisite for the commercial viability of a process [5]–[6].

Whole cells have some NAD^+^ and NADP^+^ reserves that provide a continuous source of cofactors [7]. Therefore, whole cells are used in many applications of dehydrogenases. Simultaneous overexpression of target enzymes and NAD(P)H regeneration enzymes in whole-cell biocatalysts has been carried out in many asymmetric reduction systems [8]–[9]. In this study, NAD^+^ regeneration by whole-cell biocatalysis was used to extend the applications of NAD(P) ^+^-dependent oxidoreductases.

Chiral acetoin (AC) is widely used to synthesize novel optically active α-hydroxyketone derivatives and liquid crystal composites [10]. Chemical syntheses or fermentations generally lead to the production of a mixture of both isomers [11]. Therefore, it is essential to find an effective biocatalytic process to produce enantiomerically pure AC. In the presence of NAD^+^, NAD-dependent (2*R*,3*R*)-2,3-butanediol dehydrogenase (BDH) can theoretically catalyze the stereospecific oxidation of (2*R*,3*R*)-2,3-butanediol (BD) and *meso*-2,3-BD to (3*R*)-AC and (3*S*)-AC, as shown in Scheme S1, respectively [12]–[14].

Similar to other dehydrogenases, BDHs require NAD^+^ and NADH as cofactors [12]–[14]. During the catalytic process, NAD^+^ is reduced to NADH, and 2,3-BD is oxidized to AC. Thus, during the course of one cycle, NAD^+^ is depleted while NADH and AC are accumulated. A cofactor regeneration process is necessary to obtain high AC productivity. In contrast to the more common NADH oxidase (NOX) that converts O_2_ to H_2_O_2_, the unique NOX from *Lactobacillus brevis* regenerates NAD^+^ from NADH by reducing O_2_ to H_2_O [15]–[18]. Since proteins are deactivated upon exposure to H_2_O_2_, the novel characteristics of the NOX from *L. brevis* make it a promising alternative for NAD^+^ regeneration. In this study, we developed a coexpression system in which acetoin reductase/2,3-BDH encoded by the *Bacillus subtilis ydjL* (*bdhA*) gene was the producing enzyme and NOX was the cofactor-regenerating enzyme. Our intention was to develop a novel biocatalytic process for the efficient production of (3*R*)-AC and (3*S*)-AC with high enantiomeric excess (ee).

## Results and Discussion

### Construction of the Whole-Cell Biocatalyst

**Figure 1:**
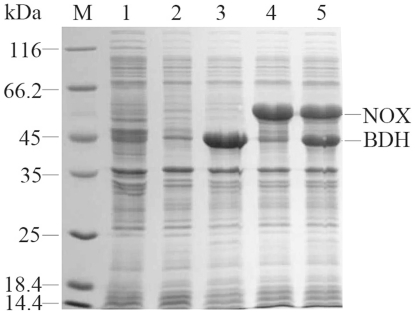
SDS-PAGE analysis of recombinant *E. coli*. Lane M, marker proteins; lane 1, *E. coli* BL21(DE3); lane 2, *E. coli* BL21(DE3) (pETDuet); lane 3, *E. coli* BL21(DE3) (pETDuet-*ydjL*); lane 4, *E. coli* BL21(DE3) (pETDuet-*nox*); and lane 5, *E. coli* BL21(DE3) (pETDuet-*ydjLnox*).

First, *Escherichia coli* BL21(DE3) (pETDuet-*ydjL*) carrying *ydjL*, which encodes (2*R*,3*R*)-2,3-BDH from *B. subtilis* 168 was constructed. The crude extract of induced *E. coli* BL21(DE3) (pETDuet-*ydjL*) cells showed a high (2*R*,3*R*)-2,3-BDH activity of 16.0 U mg^−1^ (in the direction of (2*R*,3*R*)-2,3-BD production). Next, *E. coli* BL21(DE3) (pETDuet-*nox*) harboring *nox*, which encodes NOX from *L. brevis*, was constructed. The crude extract of induced *E. coli* BL21(DE3) (pETDuet-*nox*) cells showed a high NOX activity of 7.2 U mg^−1^ (SDS-PAGE analyses of the recombinant proteins are shown in Fig. 1). The biocatalytic activities of the crude extracts prepared from *E. coli* BL21(DE3) (pETDuet-*nox*) and *E. coli* BL21(DE3) (pETDuet-*ydjL*) cells were assayed using *meso*-2,3-BD and (2*R*,3*R*)-2,3-BD as the substrates. However, no notable production of AC was detected.

In comparison with free enzymes, whole-cell biocatalysts are much more convenient to use and less expensive to prepare [19]. Enzymes in whole cells are more stable than isolated enzymes because these are protected from the external environment. The biocatalytic activities of the whole-cell biocatalyst prepared from *E. coli* BL21(DE3) (pETDuet-*ydjL*) were determined using *meso*-2,3-BD, (2*R*,3*R*)-2,3-BD, and (2*S*,3*S*)-2,3-BD as the substrates. As shown in Table 1, the whole-cell biocatalyst of *E. coli* BL21(DE3) (pETDuet-*ydjL*) had almost the same biocatalytic activities on *meso*-2,3-BD and (2*R*,3*R*)-2,3-BD but had no activity on (2*S*,3*S*)-2,3-BD.

**Table 1:** The whole-cell biocatalytic activity of recombinant *E. coli* with 2,3-BD.

*E. coli* strain	Whole-cell biocatalytic ability (U mL^−1^)		
	*meso*-2,3-BD	(2*R*,3*R*)-2,3-BD	(2*S*,3*S*)-2,3-BD
BL21 (DE3) (pETDuet)	0.3 (±0.00)	0.3 (±0.07)	ND
BL21 (DE3) (pETDuet-*ydjL*)	8.1 (±0.91)	8.9 (±0.74)	ND
BL21 (DE3) (pETDuet-*ydjL*) and BL21 (DE3) (pETDuet-*nox*)	7.71 (±0.00)	8.02 (±0.56)	ND
BL21 (DE3) (pETDuet-*ydjLnox*)	15.8 (±1.24)	14.4 (±1.24)	ND

ND: not detected.

To obtain high AC productivity, we decided to couple a cofactor regeneration system, in which biocatalysis was performed using whole cells of both *E. coli* BL21(DE3) (pETDuet-*ydjL*) and *E. coli* BL21(DE3) (pETDuet-*nox*) as catalysts. However, no enhancement of the biocatalytic activity of *E. coli* BL21(DE3) (pETDuet-*ydjL*) system was detected with supplemented whole cells of *E. coli* BL21(DE3) (pETDuet-*nox*). This may be due to the fact that (2*R*,3*R*)-2,3-BDH and NOX are physically separated and expressed in different cells, and the cellular membrane of the cells might retard the exchange of pyridine cofactors between *E. coli* BL21(DE3) (pETDuet-*nox*) and *E. coli* BL21(DE3) (pETDuet-*ydjL*). To validate this observation, *E. coli* BL21(DE3) (pETDuet-*ydjLnox*) cells were constructed in which NOX, the NAD^+^ regeneration enzyme, was coexpressed with (2*R*,3*R*)-2,3-BDH, the catalyzing enzyme, as shown in Fig. 1.

**Figure 2:**
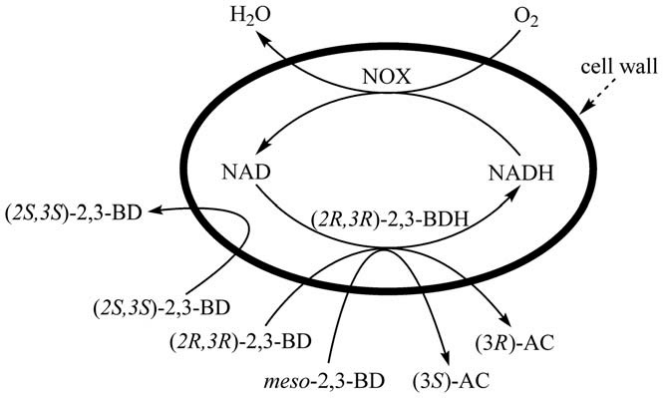
A recombinant *E. coli* whole-cell biocatalyst with BDH and the enzymatic NAD regeneration system for the production of chiral AC and (2*S*,3*S*)-2,3-BD.

Although the two genes were coexpressed on one expression vector, they were placed under two separate T7 lac promoters so that the expression of one gene would not affect that of the other. NOX could oxidize the NADH produced by (2*R*,3*R*)-2,3-BDH and provide the oxidized form of the pyridine nucleotide cofactor for the biocatalytic process, without any hindrance from the transmembrane process. As shown in Table 1, the whole-cell biocatalytic activity of *E. coli* BL21(DE3) (pETDuet-*ydjLnox*) on *meso*-2,3-BD (15.8 U mL^−1^) was about twice that of *E. coli* BL21(DE3) (pETDuet-*ydjL*) (8.1 U mL^−1^). The results indicated that recombinant *E. coli* BL21(DE3) (pETDuet-*ydjLnox*) could increase biocatalytic efficiency of BDH by co-expressing an NAD^+^ regeneration enzyme. Production of (3*S*)-AC, (3*R*)-AC, and (2*S*,3*S*)-2,3-BD was possible using *E. coli* BL21(DE3) (pETDuet-*ydjLnox*) as the biocatalyst and 2,3-BD as the substrate, as shown in Fig. 2.

### Effects of pH and Substrate Concentration on Chiral AC Biosynthesis

**Figure 3:**
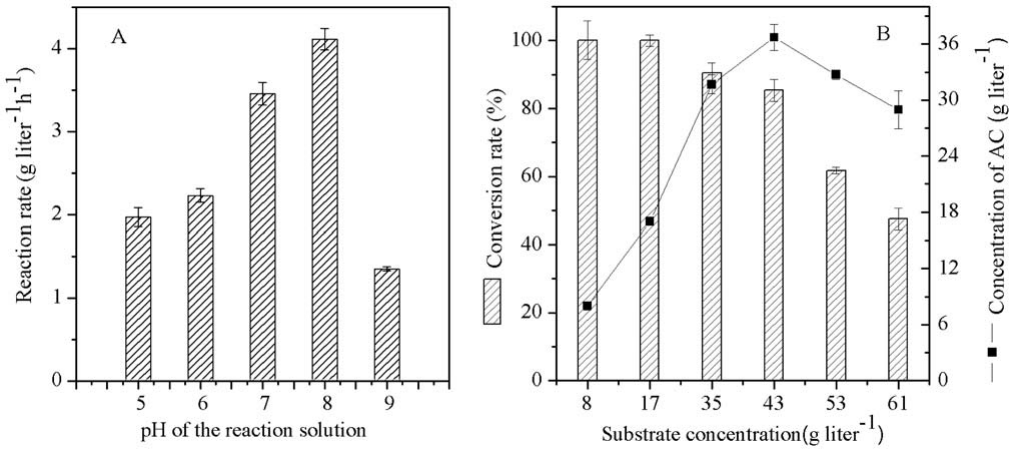
Effects of biocatalysis conditions on the reaction rate. A. pH; B. substrate concentration. *E. coli* BL21(DE3) (pETDuet-*ydjLnox*) was incubated with *meso*-2,3-BD in the reaction.

To achieve high product concentrations, the effect of pH on the yield of AC from 2,3-BD was investigated. As shown in Fig. 3A, reactions with 43.0 g L^−1^ of *meso*-2,3-BD as the substrate and 5.0 g dry cell weight (DCW) L^−1^ of *E. coli* BL21(DE3) (pETDuet-*ydjLnox*) as the biocatalyst were carried out under different conditions. After 6 h of reaction with *E. coli* BL21(DE3) (pETDuet-*ydjLnox*), the reaction rates were measured by monitoring the yield of (3*S*)-AC. The results showed that the reaction rate was the highest at pH 8.0. It was reported that the optimum pH value for the conversion of 2,3-BD to AC by (2*R*,3*R*)-2,3-BDH was 9.0 and 5.5∼7.0 for NOX [13], [18]. When the two enzymes were coexpressed and functioned together, the optimum pH value was 8.0.

Substrate concentration is another important parameter in the catalytic process, and it requires careful investigation because high substrate concentrations may lead to substrate inhibition. The effect of the substrate concentration on the conversion rate was tested (Fig. 3B). The results showed that in the range 8∼61 g L^−1^, the conversion rate of *meso*-2,3-BD decreased sharply from 100% to 47.5% as the concentration increased. Simultaneously, the concentration of (3*S*)-AC increased linearly with the increase in the substrate concentration from 8 g L^−1^ to 43 g L^−1^, but when the substrate concentration increased beyond 43 g L^−1^, the concentration of (3*S*)-AC decreased rapidly. It is known that AC can be easily separated from the reaction solution due to its low boiling point and that residual 2,3-BD can be reused. Therefore, 43 g L^−1^ of *meso*-2,3-BD was selected as a suitable substrate concentration based on the final yield of (3*S*)-AC.

### Production of Chiral AC by Recombinant *E. coli*

**Figure 4:**
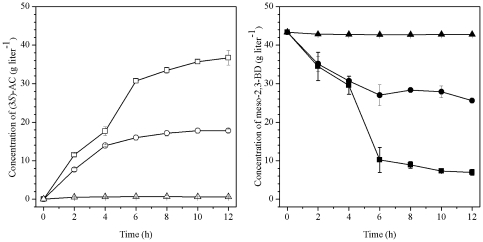
Time course of the recombinant *E. coli* whole-cell biocatalyst-mediated (3*S*)-AC production from *meso*-2,3-BD by the recombined *E. coli*. The concentration of (3*S*)-AC (□) and *meso*-2,3-BD (▪) in *E. coli* BL21(DE3) (pETDuet-*ydjLnox*) ; concentrations of (3*S*)-AC (○) and *meso*-2,3-BD (•) in *E. coli* BL21(DE3) (pETDuet-*ydjL*); and concentrations of (3*S*)-AC (▵) and *meso*-2,3-BD (▴) in *E. coli* BL21(DE3).

**Figure 5:**
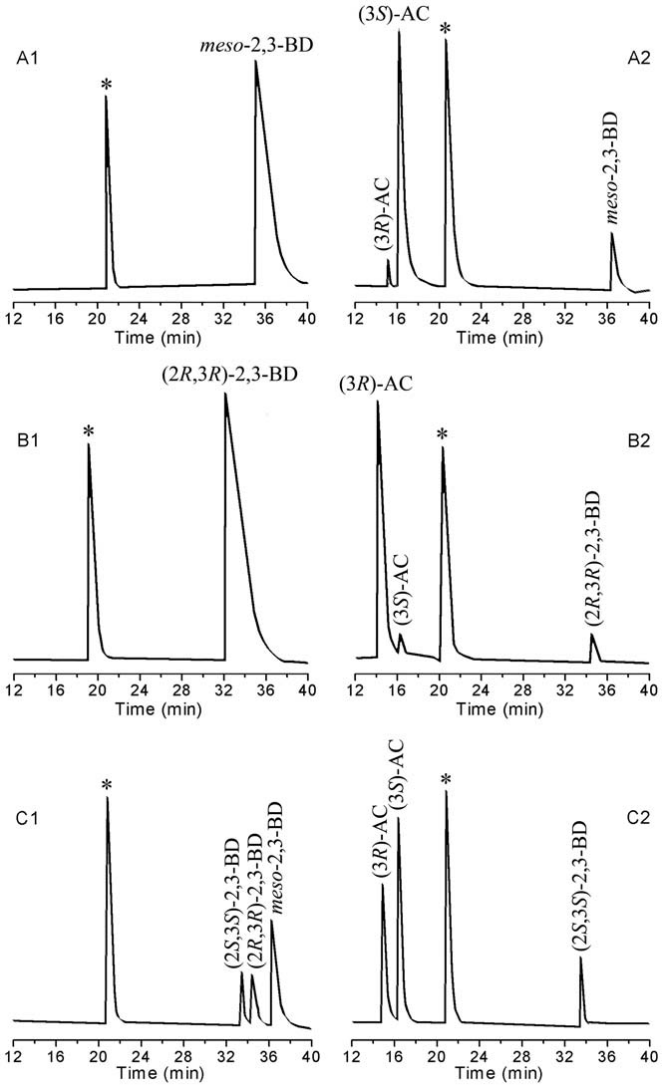
GC analyses of substrates and products of the catalytic reaction (* Isoamyl alcohol was used as the internal standard). A. Conversion of *meso*-2,3-BD to (3*S*)-AC: A1-before the reaction; A2-after the reaction; B. Conversion of (2*R*,3*R*)-2,3-BD to (3*R*)-AC: B1-before the reaction; B2-after the reaction; C. The starting material and products of kinetic resolution from a mixture of 2,3-BD: C1-before the reaction; C2-after the reaction.

To evaluate the efficiency of whole-cell in the biosynthesis of chiral AC, recombinants *E. coli* BL21(DE3) (pETDuet-*ydjLnox*) and *E. coli* BL21(DE3) (pETDuet-*ydjL*) were used to catalyze the conversion of *meso*-2,3-BD to (3*S*)-AC (*E. coli* BL21(DE3) was used as the control). As shown in Fig. 4, under optimal reaction conditions, the final concentration of (3*S*)-AC produced by recombinant *E. coli* BL21(DE3) (pETDuet-*ydjLnox*) was 36.7 g L^−1^ from 43 g L^−1^ of *meso*-2,3-BD in 12 h. In comparison, only 17.8 g L^−1^ of (3*S*)-AC was produced by recombinant *E. coli* BL21(DE3) (pETDuet-*ydjL*) under identical conditions. Low conversion rate was observed by the wild-type *E. coli* BL21(DE3) strain, and the final concentration of (3*S*)-AC was only 0.6 g L^−1^. When (2*R*,3*R*)-2,3-BD was used as the substrate, (3*R*)-AC was produced. The highest concentration of (3*R*)-AC (41.8 g L^−1^) was obtained when the concentration of (2*R*,3*R*)-2,3-BD was 43 g L^−1^. Since (3*S*)-AC and (3*R*)-AC standards could be separated by GC, the ee of the products was calculated by the amount of each isomer. The two isomers of AC were produced in 96.0% ee. The results from GC analyses of substrates and products of the catalytic reaction are shown in Fig. 5A, B.

The production of chiral AC by biocatalysis has been reported earlier [20]–[21]. Yamada-Onodera et al. reported that about 9.68 g L^−1^ of both (3*R*)-AC from (2*R*,3*R*)-2,3-BD and (3*S*)-AC from *meso*-2,3-BD were obtained after 24 h of incubation with recombinant *E. coli* expressing only glycerol dehydrogenase (GDH) [20]. In this study, a recombinant strain that coexpressed (2*R*,3*R*)-2,3-BDH and NOX was constructed, and the yield of both (3*S*)-AC and (3*R*)-AC was found to be much higher than that in previous reports. The results also showed that the recombinant strain that coexpressed (2*R*,3*R*)-2,3-BDH and NOX was significantly more efficient than the recombinant strain that only expressed (2*R*,3*R*)-2,3-BDH in terms of AC production. As shown in Fig. 4, the yield of (3*S*)-AC from the two-enzyme system was 42.8% higher than that from the one-enzyme system (similar result was found for (3*R*)-AC production, data not shown).

### (2*S*,3*S*)-2,3-BD Production by Kinetic Resolution

2,3-BD, the hydrogenation product of AC, is not only used as a fuel, but also as a valuable chemical feedstock [22]–[23]. (2*S*,3*S*)-2,3-BD can also be used as an antifreeze agent due to its low freezing point (−60°C) [22]–[23]. However, to date, no microorganism capable of producing enantiopure (2*S*,3*S*)-2,3-BD has been identified. In this study, (2*S*,3*S*)-2,3-BD was produced by kinetic resolution from a mixture of 2,3-BD using the whole-cell biocatalyst of *E. coli* BL21(DE3) (pETDuet-*ydjLnox*). When a mixture of 20.0 g L^−1^ 2,3-BD was incubated with *E. coli* BL21(DE3) (pETDuet-*ydjLnox*) at pH 8.0 and 37°C, 2.4 g L^−1^ of (2*S*,3*S*)-2,3-BD and 17.2 g L^−1^ of AC were produced in 10 h, and the ee of the (2*S*,3*S*)-2,3-BD finally obtained was higher than 99.0%. The results from GC analyses of substrates and products of the catalytic reaction are shown in Fig. 5C.

Although the yield of (2*S*,3*S*)-2,3-BD was not very high due to the low content of (2*S*,3*S*)-2,3-BD in the mixture of 2,3-BD (only 12.4%), the biocatalytic efficiency of the (2*R*,3*R*)-2,3-BDH and NOX coexpressing recombinant strain was much higher than that of recombinant *E. coli* expressing GDH only (1.4 g L^−1^) [20]. This shows that the incorporation of a cofactor regeneration system in the *E. coli* whole-cell biocatalyst is a powerful strategy for enhancing the catalytic efficiency of oxidoreductases.

Many dehydrogenases that are useful in industrial production are cofactor-dependent. By using a suitable enantioselective dehydrogenase, important enantiomerically pure compounds can be prepared by kinetic resolution. For example, NAD^+^ dependent (*S*)-ADH can be used for the production of (*R*)-phenylethanol from a racemate with 100% ee and 100% yield. Regeneration of the cofactor is important in large-scale applications of coenzyme-dependent reactions [24]. NAD(P)H regeneration has been widely studied, whereas NAD(P)^+^ regeneration, which is required for reactions involving the oxidized forms of the pyridine nucleotide cofactors, is less well developed [15]. An NAD-dependent alcohol dehydrogenase from *L. brevis* coupled with the NOX from *Lactobacillus sanfranciscensis* was successfully used to produce enantiomerically pure alcohol from a racemic mixture [15]. The two enzymes were first purified, and then coupled in a reaction in which NOX was used to regenerate the oxidized cofactor. Although this method did not generate any byproducts, the complex purification processes and the oxygen sensitivity of NOX precluded its applications on a preparative scale [3].

In comparison with isolated enzymes, utilization of whole-cell biocatalysts circumvents laborious protein purification steps, which simplifies the reactions in many cases. NAD(P)^+^ regeneration in whole-cell biocatalysts would help in extending the applications of NAD(P)-dependent oxidoreductases. In this study, we constructed a novel whole-cell biocatalyst from recombinant *E. coli* in which (2*R*,3*R*)-2,3-BDH and NOX were coexpressed to catalyze the production of (3*R*)-AC, (3*S*)-AC, and (2*S*,3*S*)-2,3-BD with high ee. The present study clearly demonstrates the importance of providing sufficient amounts of the oxidized form of the pyridine nucleotide cofactors to NAD-dependent dehydrogenases. The value of the NAD^+^ regeneration technique presented in this paper could extend beyond chiral AC and (2*S*,3*S*)-2,3-BD production to other chiral chemicals. Coupling NOX from *L. brevis* in the biocatalytic system provided a suitable method for NAD^+^ regeneration, and this should open up the possibilities for constructing other whole-cell biocatalysts with different NAD-dependent dehydrogenases.

## Materials and Methods

### Chemicals

*Meso*-2,3-BD (98.0%) and (2*R*,3*R*)-2,3-BD (98.0%) were purchased from ACROS. The mixture of 2,3-BD (75.0% *meso*-2,3-BD, 12.6% (2*R*,3*R*)-2,3-BD, and 12.4% (2*S*,3*S*)-2,3-BD) was obtained from Sinopharm (Beijing, China). Ampicillin and NADH were from Amresco. Isopropyl-β-D-1-thiogalactopyranoside (IPTG) and phenylmethanesulfonyl fluoride (PMSF) were from Merck. All other chemicals were commercially available reagents of analytical grade. PCR primers were prepared by Sangon (Shanghai, China). *Pfu* DNA polymerase was obtained from Promega (USA). T4 DNA ligase and restriction endonucleases were obtained from Fermentas (Lithuania).

### Constructions of *E. coli* BL21(DE3) (pETDuet-*ydjL*), *E. coli* BL21(DE3) (pETDuet-*nox*) and *E. coli* BL21(DE3) (pETDuet-*ydjLnox*)

The bacterial strains and plasmids used in this study are listed in Table 2. *B. subtilis* 168 genomic DNA was extracted with the Wizard Genomic DNA Purification Kit (Promega, Madison, WI, USA). The *ydjL* gene was amplified by PCR using forward primer py1 with an *Nco*Ι restriction site insertion and reverse primer py2 with a *Sal*Ι restriction site insertion. The PCR product was firstly ligated to the pEasy-Blunt vector, and the resulting plasmid was designated pEasy-Blunt-*ydjL*. The pEasy-Blunt-*ydjL* was then sequenced (Sangon, Shanghai, China) to verify that no mutations were introduced by PCR. Next, to construct the recombinant plasmid pETDuet-*ydjL* under the control of the T7 promoter, pEasy-Blunt-*ydjL* was digested with *Nco*Ι and *Sal*Ι, and the gel-purified *ydjL* fragment was ligated to the pETDuet-1 vector that had been digested with the same restriction enzymes. Using the same process that described above, the *nox* gene fragment was obtained from the genome of *L. brevis* using primers pn1 (with the *Bgl*II restriction site) and pn2 (with the *Xho*Ι restriction site). To construct a coexpression system carrying *ydjL* and *nox*, pEasy-Blunt-*nox* was digested with *Bgl*II and *Xho*Ι, and the gel-purified *nox* fragment was ligated to pETDuet-*ydjL* digested with the same restriction enzymes. *E. coli* DH5α was used for general cloning, and *E. coli* BL21(DE3) was used for protein expression. Luria-Bertani (LB) medium was used for both *E. coli* and *B. subtilis* culture. DeMan-Rogosa Sharpe (MRS) medium was used for *L. brevis* culture.

**Table 2:** Strains, plasmids, and primers used in this study.

Bacterium, plasmid or primer	Genotype, properties or sequence	Source or reference
Strain		
*L. brevis*	Wild type lactic acid fermentation	CICC 6004
*B. subtilis* 168	Wild type	ATCC 23857
*E. coli* DH5α	*supE44 Δ lacU169 (ϕ80 lacZΔM15) hsdR17 recA1 endA1 gyrA96 thi-1 relA1*	Novagen
*E. coli* BL21 (DE3)	*F^−^ ompT hsdS_B_ (r_B_^−^m_B_^−^) gal (λ c I 857 ind1 Sam7 nin5 lacUV5 T7gene1) dcm (DE3)*	Novagen
Plasmid		
pEasy-Blunt	Cloning vector; Amp^r^	Transgene
pETDuet-1	Overexpression vector; Amp^r^	Novagen
pETDuet-*ydjL*	*ydjL* in pETDuet-1	This study
pETDuet-*nox*	*nox* in pETDuet-1	This study
pETDuet-*ydjLnox*	*ydjL* and *nox* in pETDuet-1	This study
Primer		
py1	5′-TCA*CCATGG*GCATGAAGGCAGCAAGA-3′	This study
py2	5′-GGC*GTCGAC*TTAGTTAGGTCTAACA-3′	This study
pn1	5′-GCG*AGATCT*CATGAAAGTCACAGTTG-3′	This study
pn2	5′-GAT*CTCGAG*TTAAGCGTTAACTGAT-3′	This study

The italic face indicates the introduction of restriction sites.

### Biocatalyst Preparation

Recombinant *E. coli* cells were grown at 37°C on a rotary shaker (180 rpm) in LB medium containing ampicillin (100 µg mL^−1^) to an OD_620nm_ value of 0.6. Expression of the recombinant gene was induced by adding 1 mM IPTG at 16°C to avoid the formation of inactive inclusion bodies. After induction, the cells were harvested by centrifugation at 6,000×*g* for 5 min at 4°C and then washed twice with 1/15 M phosphate buffer (PB) (pH 7.4). The cell pellet was resuspended in 1/15 M PB (pH 7.4), and maintained at 4°C for further studies.

### (2*R*,3*R*)-2,3-BDH and NOX Activity Assays

The harvested cells were resuspended in 20% glycerol, 0.1 mM PMSF, and 1 mM DTT in 1/15 M PB (pH 7.4) and disrupted for 20 min (5 s + 5 s) using a sonicator. The disrupted cells were centrifuged for 10 min at 10,000×*g* at 4°C, and the supernatant was used as the crude cell extract. The (2*R*,3*R*)-2,3-BDH and NOX activities were assayed by measuring the decrease in NADH (0.2 mM) at 340 nm using a UV/visible spectrophotometer (Ultrospec 2100 pro, Amersham Biosciences, USA) [18], [25]. The protein concentration was determined by the Bradford method using bovine serum albumin as the standard [26].

### Assay of Whole-Cell Biocatalytic Activity

The whole-cell biocatalytic activity was assayed by measuring the increase of AC in the reaction solution. The reaction solution consisting of 10.0 g L^−1^ 2,3-BD and the whole-cell biocatalyst (the final concentration was 1.5 g DCW L^−1^) in 200 mM PB (pH 8.0) was incubated at 37°C on a rotary shaker for 20 min. The mixture was centrifuged at 6,000×*g* for 5 min to stop the reaction, and the AC concentration in the supernatant was measured. One unit of whole-cell biocatalytic activity was defined as the amount of cells that catalyzed the formation of 1.0 µmol of AC per minute at 37°C.

### Biocatalysis Conditions

Reaction with 20 mL of mixture in a 250-mL Erlenmeyer flask at 37°C and 200 rpm were performed at pH 8.0. The cell concentration in the reaction was 5.0 g DCW L^−1^. To produce chiral AC, the harvested biocatalyst was resuspended in 200 mM PB (pH 8.0) containing 43 g L^−1^ *meso*-2,3-BD or (2*R*,3*R*)-2,3-BD. For the kinetic resolution of (2*S*,3*S*)-2,3-BD from the 2,3-BD mixture, the harvested biocatalyst was resuspended in 200 mM PB (pH 8.0) containing 20 g L^−1^ of 2,3-BD.

### Product Analysis

Samples (0.15 mL) were taken periodically and centrifuged at 15,000×*g*. The concentrations of 2,3-BD and AC in the supernatant were analyzed by GC. Prior to GC analysis, the supernatant was extracted with an equal volume of ethyl acetate after the addition of isoamyl alcohol as the internal standard. The GC (Agilent GC6820) system consisted of a flame ionization detector and a fused silica capillary column (Supelco Beta DEX™ 120, inside diameter, 0.25 mm; length, 30 m). The operating conditions were as follows. Nitrogen was used as the carrier gas. The injector temperature and detector temperature were both 280°C. The column oven was maintained at 40°C for 3 min and then programmed to increase to 80°C at a rate of 1.5°C min^−1^. The temperature was then raised to 86°C at a rate of 0.5°C min^−1^ and finally to 200°C at a rate of 30°C min^−1^. The injection volume was 3 µL. In this study, the ee values of (3*S*)-AC and (3*R*)-AC were calculated as 
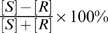
 and 
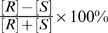
, respectively, where [*R*] and [*S*] represent the concentrations of (3*R*)-AC and (3*S*)-AC, respectively.

## Supporting Information

Scheme S1 The reactions catalyzed by (2R,3R)-2,3-butanediol dehydrogenase.
(0.27 MB TIF)
