# Supplementary figures and images for: A Novel Whole-Cell Biocatalyst with NAD+ Regeneration for Production of Chiral Chemicals
Source: PLoS One. 2010 Jan 26;5(1):e8860. doi: 10.1371/journal.pone.0008860 (PMC2811184; doi:10.1371/journal.pone.0008860)

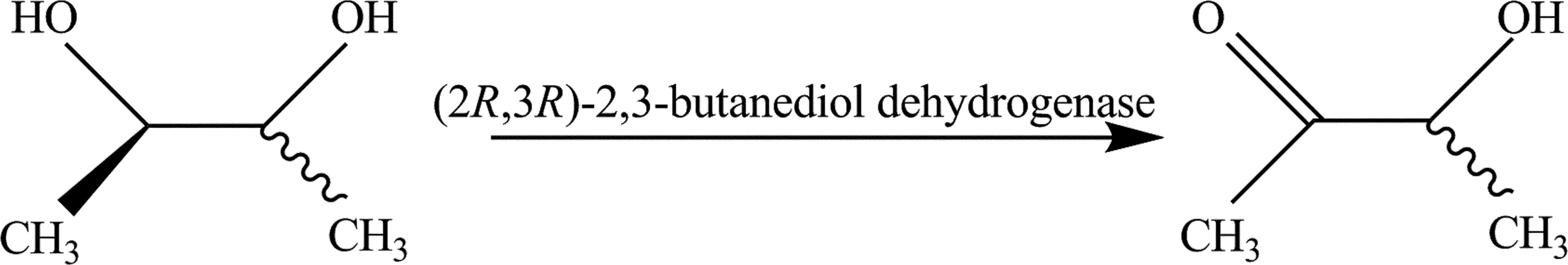

Supplement: Scheme S1 — The reactions catalyzed by (2R,3R)-2,3-butanediol dehydrogenase. (0.27 MB TIF) [file pone.0008860.s001.tif]
